# Synthesis of Hydroxyapatite with Antibacterial Properties Using a Microwave-Assisted Combustion Method

**DOI:** 10.1038/s41598-019-40488-8

**Published:** 2019-03-08

**Authors:** Suphatchaya Lamkhao, Manlika Phaya, Chutima Jansakun, Nopakarn Chandet, Kriangkrai Thongkorn, Gobwute Rujijanagul, Phuwadol Bangrak, Chamnan Randorn

**Affiliations:** 1 Master’s Degree Program in Chemistry, Faculty of Science, Chiang Mai University, Chiang Mai, 50200 Thailand; 2 PhD Degree Program in Environmental Science, Environmental Science Research Center, Faculty of Science, Chiang Mai University, Chiang Mai, 50200 Thailand; 3 School of Allied Health Sciences and Research Institute for Health Sciences, Walailak University, Nakhon Si Thammarat, 80160 Thailand; 4 Department of Chemistry, Faculty of Science, Chiang Mai University, Chiang Mai, 50200 Thailand; 5 Department of Companion Animal and wildlife clinic, Faculty of Veterinary Medicine, Chiang Mai University, Chiang Mai, 50100 Thailand; 6 Department of Physics and Materials Science, Faculty of Science, Chiang Mai University, Chiang Mai, 50200 Thailand; 7 School of Science, Walailak University, Nakhon Si Thammarat, 80160 Thailand; 8 Environmental Science Research Center (ESRC), Faculty of Science, Chiang Mai University, Chiang Mai, 50200 Thailand

## Abstract

The prevention of implant-associated infections has been increasing clinically in orthopedic surgery. Hydroxyapatite with antibacterial properties was synthesized using a microwave-assisted combustion method. High crystallinity at low temperature can be achieved using this method. The synthesized hydroxyapatite exhibited a superior clear zone for both Gram-positive and Gram-negative bacteria. Electron spin resonance (ESR) and X-ray photoelectron spectroscopy (XPS) were used for the radical investigation. The application of intelligent ink testing and an antioxidant assay using DPPH reduction were also used to confirm the existence of radicals. These techniques provided data confirming that radicals are responsible for the antibacterial properties. The synthesized antibacterial hydroxyapatite would be a good candidate for the prevention any infection with medical implants and injection materials causing failure in bone repair.

## Introduction

Biocompatible chemically-synthesized hydroxyapatite (HA) is extensively used in bone treatments, controlled drug release and dental implants^1,2^. HA coatings on metal alloy substrates have gained much attention for bone replacement because they exhibit a high biocompatibility and acceptable mechanical properties, such as a high bond strength and an elastic modulus value close to that of the bone. Incorporating hydroxyapatite into hydrogels can also be used for application in bone-like materials, and thus, it can be used in orthopedic patients^3–6^. An injectable formulation of biocompatible hydrogel/HA composite materials would reduce the trauma and surgery cost for the patients^7–10^.

Nevertheless, infection with medical implants and injection materials are still one of common causes of failure for bone repair^11–13^. The fabrication of HA composite materials with antibacterial properties could be an intriguing solution. It was found that nanoparticle composites could inhibit bacterial and fungal growth^14–17^. The incorporation of HA/hydrogel and antibiotics is also used for the inhibition of bacterial growth^8^. The effective duration has been prolonged from 24 to 72 h after soaking antibiotic-loaded HA samples in human plasma^18^. Another promising approach is the addition of metal nanoparticles as an alternative to antibiotics because of the increasing bacteria population exhibiting resistance to these drugs, consequently reducing their applicability. Silver nanoparticles have been shown to be effective to combat bacteria, viruses and eukaryotic microorganisms^16^. A HA/Ag composite material with antibacterial properties has been synthesized using thermal spray technology. This material also shows good biocompatibility and osteoconductivity, as validated by its *in vitro* properties^19^. Titanium implants coated with silver (Ag) were also investigated for their antibacterial properties. Ag incorporated into calcium phosphate (CaP) coatings on titanium (Ti) via a hydrothermal method^20^ and Ag deposited on anodized titanium (Ti)using electrophoresis^21^ were reported. It was found that the antibacterial properties of the coatings were distinctly improved by the incorporation of Ag, but the cell proliferation and differentiation were significantly decreased. However, there are some concerns with regards to the cytotoxicity of Ag, and other dopants have been explored as alternative dopants or binary dopants to reduce the cytotoxicity of Ag. Adding strontium (Sr) as a binary dopant in the coatings allowed the negative effects of Ag to be reduced while maintaining good antibacterial properties^20^. Cerium-substituted hydroxyapatite (CeHAP) synthesized by a sol-gel method exhibited antibacterial properties as well, but the activity depends on the concentration of cerium ions. The cerium ion concentration plays a key role in determining the antibacterial properties^22^, which is similar to Ag. The Ag concentration also affects osteoblast cell attachment^23^. The incorporation of Mg^2+^, Ni^2+^, and SiO_4_ ions into the hydroxyapatite (HA) structure also showed *in vitro* antibacterial activity against *E*. *coli* and *P*. *Aeruginosa*^24,25^. The antibacterial properties of the HA/Ag composite material may be improved by doping other metal ions such as Zn, Ti, or Cu ions^26,27^.

However, the incorporation of nanoparticles may lead to some long-term harmful side effects, whereas, antibiotics might not be active long enough^16,17,19,28^. HA with antibacterial properties would be a promising material for application of HA. In general, HA does not show antibacterial properties. Therefore, a novel method to prepare antibacterial HA needs to be developed.

There are several methods for the preparation of HA such as co-precipitation, sol-gel, solid state and hydrothermal methods^29^. Currently, the microwave (MW)-assisted method is gaining much attention for material preparation because it generates heat inside the molecule compared to external heating and the subsequent radiative transfer of conventional heating^30^. The application of microwave heating to the synthesis of materials was found to improve the antibacterial properties of nanoparticles, probably because of the smaller size that can be obtained^26,31,32^. However, the mechanism needs to be further investigated.

In this work, a new multifunctional HA with antibacterial properties will be synthesized using a microwave-assisted peroxide based route, which is simple, has a low calcination temperature and is suitable to be scaled up for a process method.

## Results and Discussion

**Figure 1:**
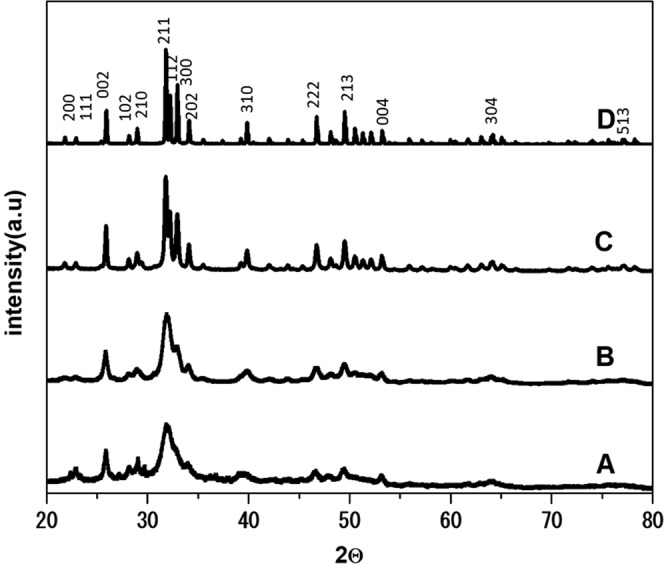
XRD patterns of commercial HA (A) and the synthesized HA by the peroxide-based route after calcination at 600 °C: filtering-HA (B), hotplate-HA (C) and microwave-HA (D).

The XRD patterns of the powder samples obtained from different synthetic methods are shown in Fig. 1. Commercial HA showed a broad HA peak (Fig. 1A) similar to the filtering-HA powders obtained by the peroxide-based route after calcination at 600 °C. Meanwhile, the XRD pattern of the microwave-HA powders obtained by the peroxide-based route after calcination at 600 °C showed a significantly sharper peak than the others. A higher temperature with some combustion occurring during evaporation was proposed for this process. The microwave-assisted synthesis of HA seems to be a superb candidate for HA synthesis, which can be achieved with a high crystallinity at low temperature.

**Figure 2:**
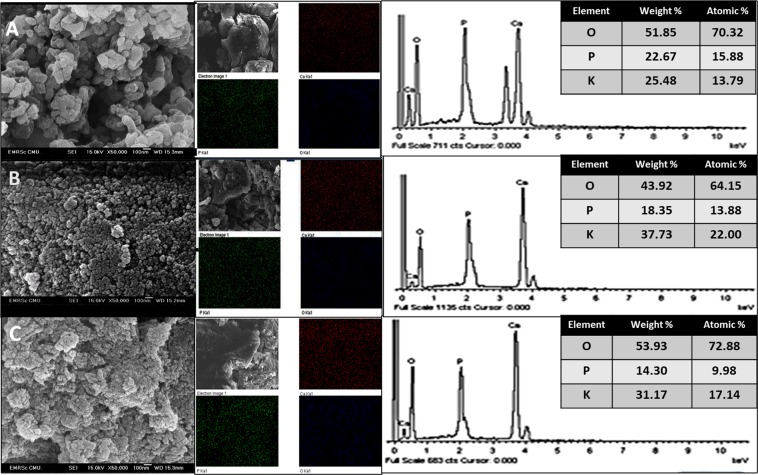
SEM-EDS images of the synthesized HA by a peroxide-based route after calcination at 600 °C: filtering-HA (**A**), microwave-HA (**B**) and hotplate-HA (**C**).

The morphologies of the powders synthesized by drying with filtering and evaporation using microwave and hotplate heating were apparently different, as shown in Fig. 2. An agglomeration of small particles was observed for the filtering-HA powder, leading to some noticeable bunches of particles of approximately 100 nm. The powders prepared by evaporating using microwave and hotplate were smaller distinct particles than filtering-HA correlating with vigorous reaction of combustion. A particle size of approximately 20–50 nm of closely arranged particles in a planetary shape with less pores can be observed. Microwave-HA powders showed less agglomeration than hotplate-HA probably because microwave radiation generates heat inside molecules and induce more dispersion of the PO_4_^3−^ ions nuclei, which possess strong polarizability and thus are excellent microwave-absorbing agents. As a result, combustion reaction of microwave-HA is more vigorous than hotplate-HA as can be noticed from the higher crystallinity XRD pattern. The Ca/P ratio and elemental mapping of the pelletized samples were obtained by an area scan using semiquantitative EDX analysis. The Ca/P ratios were 1.72, 1.73 and 1.64 for the pellets obtained from the filtering-HA, hotplate-HA and microwave-HA, respectively. The resulting ratios correlated with the theoretical value of 1.67 and corresponded to the XRD patterns. The elemental map of the calcium, phosphorus and oxygen showed a good distribution throughout the pellets.

**Figure 3:**
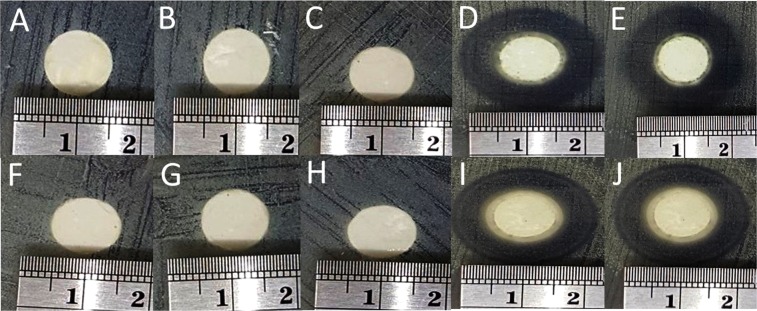
Images of the antibacterial properties of HA on *E. coli:* (**A**) commercial-HA, (**B**) filtering-HA using H_2_O_2_, (**C**) filtering-HA, (**D**) microwave-HA using H_2_O_2_, and (**E**) microwave-HA; and on S-Aureus: (**F**) commercial-HA, (**G**) filtering-HA using H_2_O_2_, (**H**) filtering-HA, (**I**) microwave-HA using H_2_O_2_, and (**G**) microwave-HA.

The antibacterial properties were validated for both Gram-positive and Gram-negative bacteria. The samples were pressed into a pellet before testing in order for the clear zone to be clearly observed. Commercial HA did not show any activity for both Gram-positive and Gram-negative bacteria (Fig. 3A,F) as well as the filtering-HA (Fig. 3B,C,G,H). Interestingly, the microwave-HA samples exhibited a superior clear zone for both Gram-positive and Gram-negative bacteria independent of the solvent used during preparation (Fig. 3D,E,I,J). It can be concluded that hydrogen peroxide was not involved in the antibacterial properties.

The antibacterial properties may be induced by nanoparticles *via* several mechanisms, e.g., alteration of the cell wall and cytoplasm, alteration of the membrane, or inhibition of the respiratory activity^33^. However, TEM showed that the filtering-HA and microwave-HA samples seem to have particles in the same size range (S1). Therefore, the particle size did not have any influence on the antibacterial activity of the microwave-HA sample. What occurred during synthesis using microwave-assisted drying might play a key role in the antibacterial activity. It was found that a huge smoke was evolved during drying using microwaves. A combustion reaction was proposed for microwave-HA synthesis. Ammonium nitrate might be formed during drying because calcium nitrate and ammonia have been added into the solution, as has been reported for TiO_2_ synthesis^34^. As a result, ammonium nitrate can act as an oxidant for the combustion reaction.

If the antibacterial properties were initiated by the combustion method, the sample prepared by drying with a hotplate should also show similar results. Moreover, the antibacterial properties of the powders, which are just dried before combustion, would be helpful to clarify the effect of combustion. Figures S2 and S3 showed the antibacterial testing of the pellets of samples comparing among drying without (S2) and with combustion (S3) for both the drying using a hotplate and microwaves. Figure S2 shows the negative results for all samples, whereas Fig. S3 shows the positive results, indicating that combustion is responsible for the antibacterial properties of the synthesized samples.

**Figure 4:**
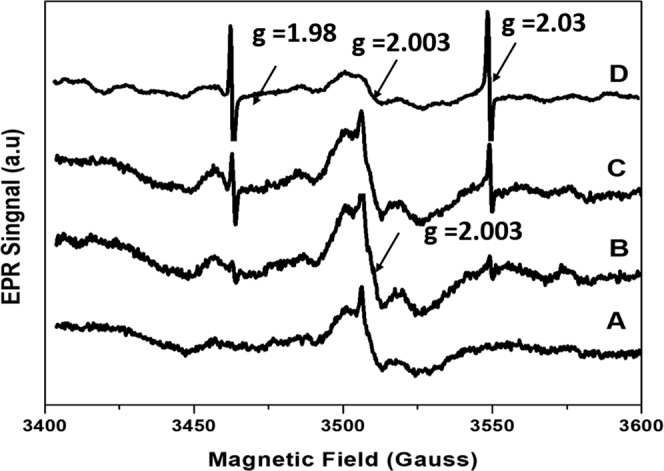
ESR signal for (A) filtering-HA after calcination at 600 °C, (B) hotplate-HA after calcination at 600 °C, (C) microwave-HA after calcination at 600 °C and (D) microwave-HA after calcination at 1,200 °C.

The investigation of the species influenced by the antibacterial properties was conducted by both an instrumental technique and experimental method. Radical species might play a key role in the antibacterial activity. ESR measurements of the samples possess negative and positive results, as shown in Fig. 4. The samples after calcination at 600 °C (Fig. 4A–C) exhibit a broad peak at g = 2.003, which may correlated to a free electron. However, a free electron should provide a sharp peak, but the samples show a broad peak, meaning that it cannot exactly be confirmed as a free electron. Interestingly, two peaks at g = 1.98 (3480G) and g = 2.03 (3550G) were obviously observed for the hotplate-HA sample after calcination at 600 °C and intensity increases dramatically for the microwave-HA sample (Fig. 4B,C). Calcination at higher temperature also affected the intensity of the two peaks, the intensity increase with increasing temperature as can be seen for microwave-HA after calcination at 600 °C and 1,200 °C in Fig. 4C,D. It is clearly seen that the two peaks can be better generated at higher temperatures. A phosphate radical or hydroxyl radical was proposed for these g values because there are 2 symmetric peaks, indicating that an ion with I = 1/2 should be the source of the peaks. A phosphate radical is probably a key species because of the large phosphorus coupling^35^. However, these *g*-values have not exactly been reported in the previous works. Three peaks at g = 2.01, 2.003 and 1.99 were found to arise from some radical-related defect of hydroxyapatite, such as peroxyl radicals or carbon dioxide radical anions (CO_2_^·−^)^36,37^. Active oxygen species on hydroxyapatite were reported at g = 2.017, 2.011, and 2.003^38^. Several active species from a defect in hydroxyapatite were also found at g = 2.0019 for a singlet of an electron at the OH vacancy and g = 2.0018 and 2.0683 for the doublet of the oxygen anion radical interacting with neighboring OH. The ESR signals at the holes of the carbonate ion and phosphate ion were observed at g = 2.0034, 2.0018, and 1.9971 and g = 2.030, 2.0062, and 2.014, respectively^39,40^. According to the similarity of ESR signals in this work and the signal from the hole at the phosphate ion, the phosphate radical seems to be an active species occurring during combustion. However, it is difficult to understand how the phosphate radical can be formed, and these results are under further investigation.

In order to confirm the effect of the radicals on the antibacterial properties, the microwave-HA and hotplate-HA samples prepared with and without the addition of H_2_O_2_ and after calcination at 1,200 °C have been tested. The antibacterial properties were still similar to the samples calcined at 600 °C (S4)

**Figure 5:**
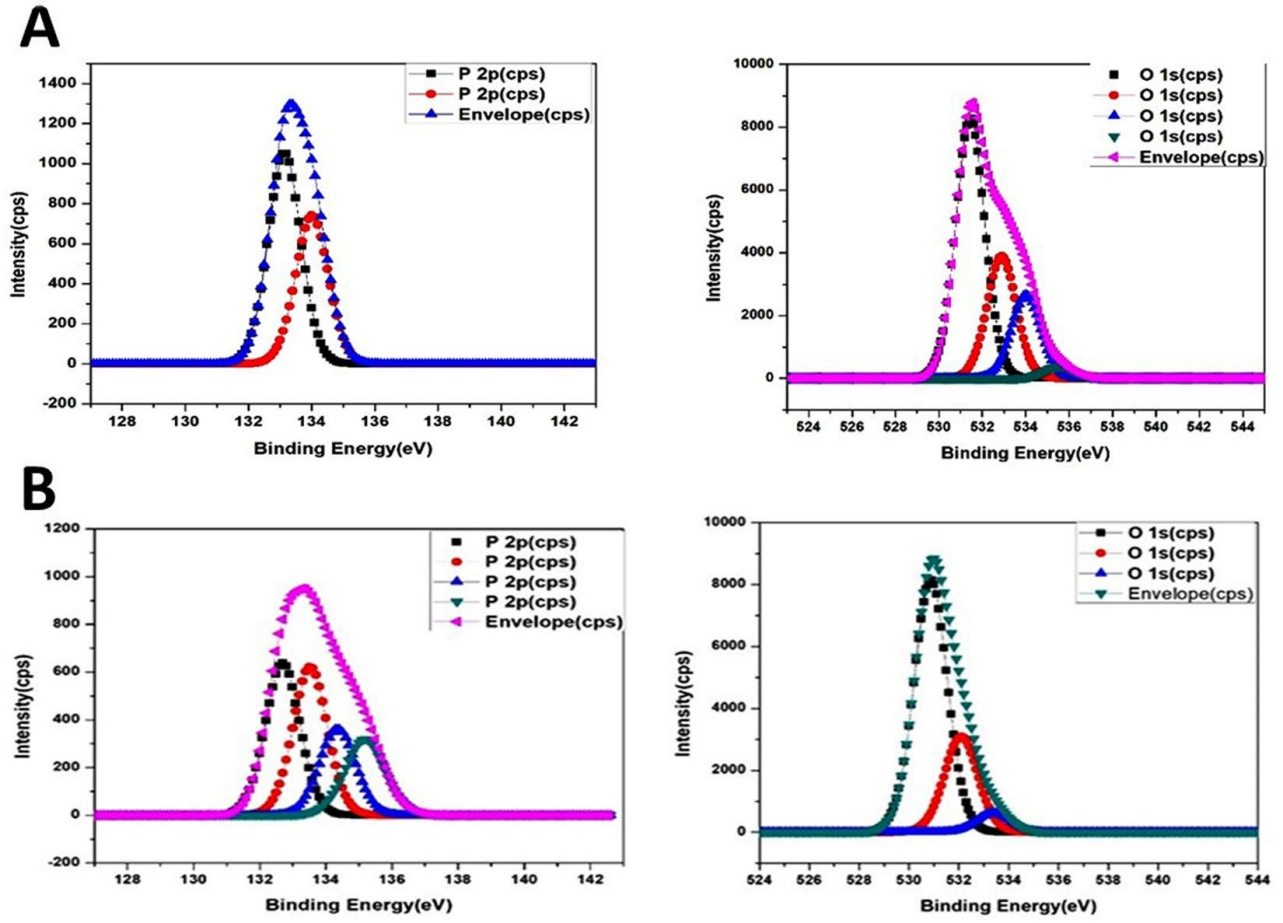
XPS spectra of (**A**) filtering-HA and (**B**) microwave-HA samples.

X-ray photoelectron spectroscopy (XPS) might be helpful to provide more information on the active ion species with regards to the antibacterial activity. Phosphorous and oxygen species were compared between the filtering-HA and microwave-HA samples, as shown in Fig. 5. It was found that there are totally different of phosphate species; two peaks were observed for the filtering-HA sample whereas four peaks can be observed for the microwave-HA sample. Different phosphate bonding may be a major source of radicals affecting the antibacterial properties.

**Figure 6:**
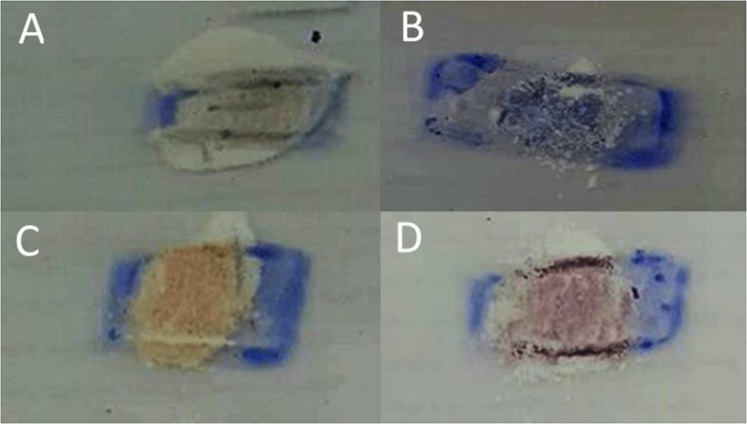
Color changes of intelligent ink when in contact with (**A**) commercial-HA, (**B**) filtering-HA, (**C**) microwave-HA and (**D**) hotplate-HA.

To confirm whether the antibacterial properties occur because of radicals, the color changing of intelligent ink, which is a commercial smart ink for the assessment of low activity photocatalytic surfaces^41^, was revealed. Assuming that the mechanism of photocatalytic activity occurred via radicals, if intelligent ink has a color change when in contact with the microwave-HA, it may be concluded that there are radicals at the surface of the microwave-HA. The explorer intelligent ink, which will change from blue to pink, was used for this study. Figure 6 shows the image of the commercial-, filtering- and microwave-HA powders put onto a band of intelligent ink-coated glass. It was found that the commercial- and filtering-HA did not affect the color of the intelligent ink, whereas, the microwave- and hotplate-HA changed the color of the intelligent ink from blue to pink. These results were related to the antibacterial properties tested, leading to the same assumption of having radicals at the surface of microwave- and hotplate-HA.

**Figure 7:**
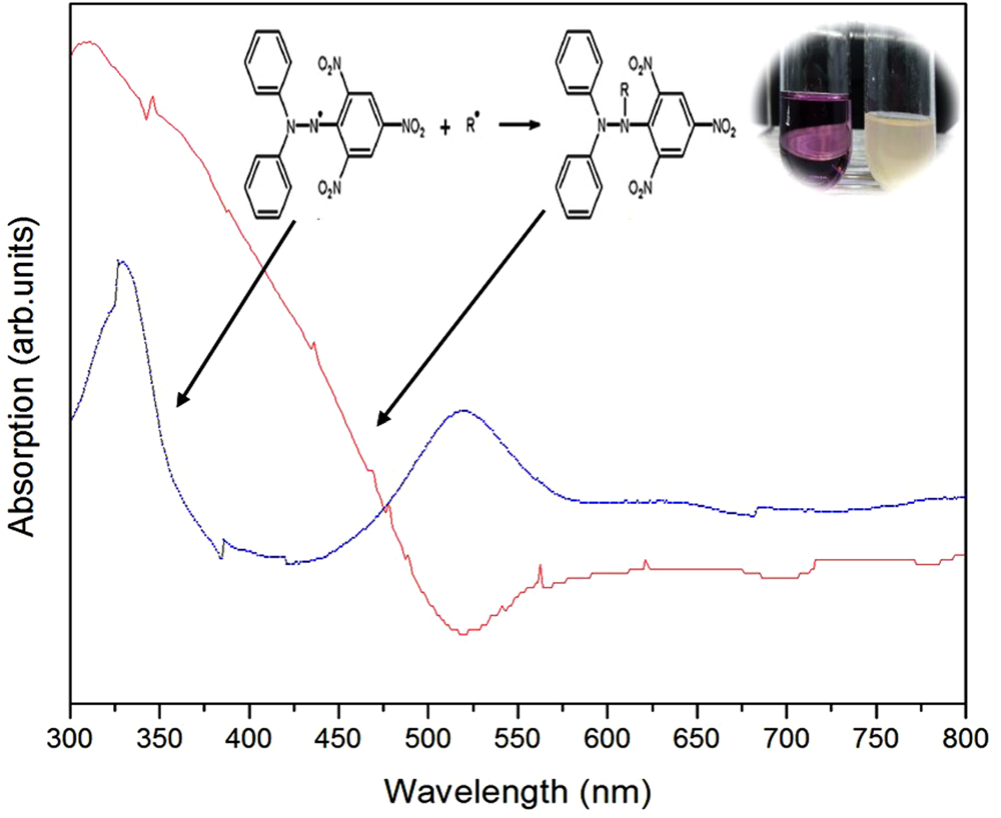
The DPPH solution changed from violet to yellow color when adding microwave-HA powder into the DPPH solution.

Moreover, the radicals occurring in microwave-HA were also examined with the antioxidant assay using DPPH reduction. It was found that the result of using microwave-HA is positive, similar to the previous results. The DPPH solution changed from a violet to yellow color, thus showing the radical scavenging activity, as shown in Fig. 7. Commercial- and filtering-HA samples showed negative results, and no color change was observed, leading to the conclusion that there are radicals on the surface of microwave-HA.

**Figure 8:**
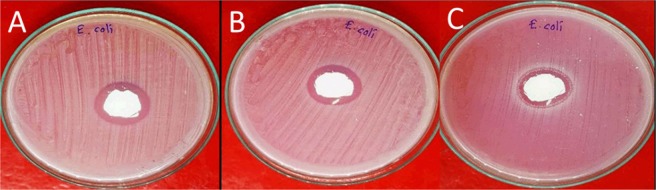
Photographs of the changes in the clear zone of antibacterial testing using microwave-HA for *E*. *Coli*: (**A**) 24 h, (**B**) 168 h, (**C**) 720 h.

The effective duration of the antibacterial activity of the microwave-HA was investigated. The changes in the clear zone of antibacterial testing for 24, 168 and 720 were photographed, as shown in Fig. 8. Interestingly, the clear zone can exist for 720 hours of testing. It can be concluded that the microwave-HA showed impressive antibacterial properties in terms of the effective duration, which has been prolonged from 24 to 720 hours.

### Cytotoxicity test

**Figure 9:**
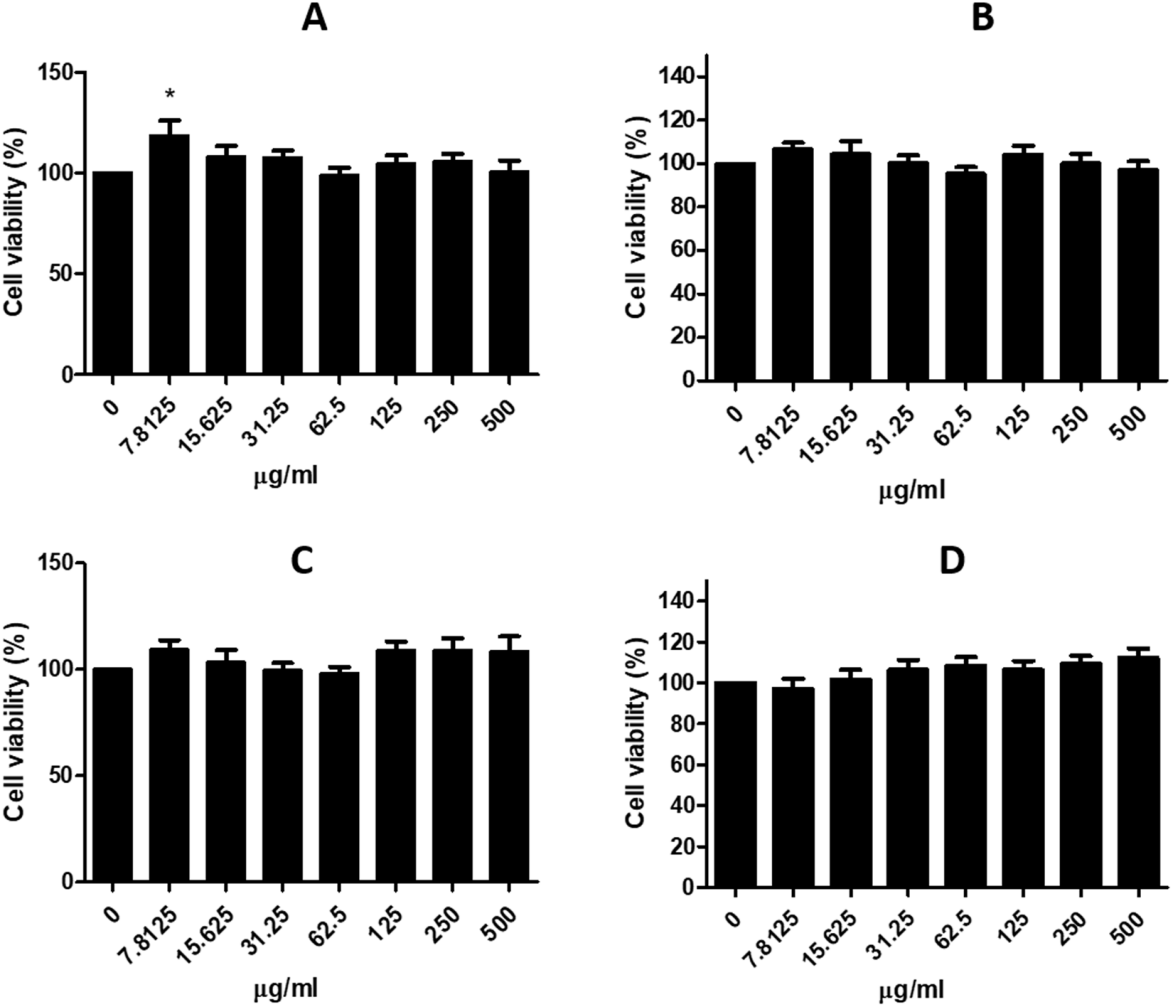
Cell viability after treatment with (**A**) commercial-HA, (**B**) filter-HA after calcination at 600 °C, (**C**) hotplate-HA after calcination at 600 °C and (**D**) microwave-HA after calcination at 600 °C at various concentrations.

Treatment of RAW 264.7 macrophages with filter-HA, hotplate-HA and microwave-HA at concentrations up to 500 μg/mL for 24 h did not affect cell viability comparable to that of the commercial HA (Fig. 9). It has been shown that synthesized HA did not exhibit any cytotoxic effect as determined by LDH measurement

## Conclusion

Hydroxyapatite with antibacterial properties can be synthesized using a microwave-assisted combustion method. ESR signals, intelligent ink testing and an antioxidant assay using DPPH reduction provided data confirming that radicals were responsible for the antibacterial properties. The synthesized antibacterial hydroxyapatite would be a good candidate for the prevention of any infection with medical implants and injection materials causing failure in bone repair because no other ions or antibiotic needs to be incorporated and no cytotoxic effect. It also showed a superior long term activity for 720 h.

## Methods

### Preparation of hydroxyapatite

Hydroxyapatites were prepared using a simple and clean method. Calcium nitrate tetrahydrate (Sigma Aldrich, 0.25 mol) was dissolved in 150 mL of 5% V/V hydrogen peroxide (Merck). A stoichiometric amount of orthophosphoric acid (RCL labscan) was then slowly added dropwise with continuous stirring into the calcium nitrate solutions. White precipitates were obtained after adjusting the pH of the solution to a pH of 10 using ammonium hydroxide (J.T. Baker). The suspensions were then continuously stirred for an additional 30 min at ambient temperature. The white calcium phosphate compound powders were separated from the solution by three different methods. First, the suspensions were separated by filtering and washing with distilled water several times (hereinafter referred to as filtering-HA). Second, the suspensions were separated by evaporating until dryness using a microwave oven (Electrolux EMM2009W) with a perforated top casing, and the solution was heated in a cycle of 5 min on and 5 min off (hereinafter referred to as microwave-HA). Third, the suspensions were separated by evaporating to dryness a using hotplate (hereinafter referred to as hotplate-HA). To clarify the effect of the hydrogen peroxide on antibacterial activities, the co-precipitation method in aqueous solution, without any hydrogen peroxide addition, of three separating methods was also examined for comparison. All of the obtained powders were then calcined at 600 °C for 2 h with a heating rate 10 °C/min.

### Characterization

The XRD data were collected using a Rigaku Mini Flex II X-ray diffractometer. Scanning electron microscopy and transmission electron microscopy were performed using a JEOL JSM-6335 F and JEOL JEM-2010 electron microscope, respectively. The chemical composition of the sample surface was investigated by an X-ray photoelectron spectrometer (AXIS ULTRADLD, Kratos Analytical, Manchester UK). The base pressure in the XPS analysis chamber was approximately 5 × 10^−9^ torr. The spectra were calibrated using the C1s line (BE = 285 eV). ESR measurements were obtained using an Elexys500 (Bruker) at room temperature, a frequency of ~9.8 GHz, a power of 2 mW, a modulation amplitude of 1 G, a center field of 3404 G, and a sweep width of 200 G.

### *In Vitro* antibacterial analyses

The antibacterial activities of the samples were observed by standard methods using *S*. *aureus* (Gram-positive bacterium) and *E*. *coli* (Gram-negative bacterium) on nutrient agar (NA) plates. The samples were sterilized at 110 °C prior to antibacterial testing. Each bacterial suspension, which was adjusted to a turbidity of 0.5 McFarland, was swabbed onto an NA plate using a sterilized cotton bud. Next, the samples were placed on the surfaces of NA covered with a tested bacterial strain. NA plates were incubated at room temperature (27 °C) for 24 h. The antibacterial activity was investigated by measuring the zone of inhibition (clear zone). The positive results were demonstrated as clear zones resulting from bacterial growth inhibition by the HA materials.

### Cytotoxicity test

A RAW 264.7 cell line (ATCC, Manassas, VA) was grown in RPMI**-**1640 medium (Corning, USA) containing 10% fetal bovine serum, 100 U/mL penicillin, 100 mg/mL streptomycin, and 2 mM stable L-glutamine. RAW 264.7 cells were maintained under standard conditions in a humidified atmosphere of 5% CO_2_ at 37 °C. The cells were grown in a 96-well plate at a density of 3.0 × 10^5^ cell/cm^2^ for 24 h. Cells were treated with the filter-HA, hotplate-HA and microwave-HA after calcination at 600 °C and commercial at various concentrations (0–500 μg/mL) for 24 h. After that, the spent medium was then removed and 200 μl of 0.5 mg/ml 3-(4,5-dimethylthiazolyl-2)-2,5-diphenyltetrazolium bromide (MTT) (Sigma-Aldrich, St. Louis, MO) solution was added into each well and incubated at 37 °C in a CO_2_ incubator for 3 h. The resulting formazan crystals were dissolved in DMSO, and the absorbance for each well was detected at 560 nm using a microplate reader (Thermo Fisher, USA). Data were obtained from three independent experiments in four replicates. Cell viability was calculated using the following equation:

$$[({{\rm{Abs}}}_{{\rm{treated}}{\rm{sample}}}/{{\rm{Abs}}}_{{\rm{untreated}}{\rm{sample}}})\times 100]$$

## Supplementary information

Synthesis of Hydroxyapatite with Antibacterial Properties Using a Microwave-Assisted Combustion Method
