# Supplementary material for: Synthesis of Hydroxyapatite with Antibacterial Properties Using a Microwave-Assisted Combustion Method
Source: Sci Rep. 2019 Mar 8;9:4015. doi: 10.1038/s41598-019-40488-8 (PMC6408465; doi:10.1038/s41598-019-40488-8)
Supplement: Supplementary file 1 — Synthesis of Hydroxyapatite with Antibacterial Properties Using a Microwave-Assisted Combustion Method [file 41598_2019_40488_MOESM1_ESM.docx]

**Supplementary data**

**Synthesis of Hydroxyapatite with Antibacterial Properties Using a Microwave-Assisted Combustion Method**

Suphatchaya Lamkhao^1^, Manlika Phaya^2^, Chutima Jansakun^3^, Nopakarn Chandet^4^, Kriangkrai Thongkorn^5^, Gobwute Rujijanagul^6^, Phuwadol Bangrak and Chamnan Randorn^4,8,*^

^1^Master’s Degree Program in Chemistry, Faculty of Science Chiang Mai University Chiang Mai 50200 Thailand

^2^PhD Degree Program in Environmental Science, Environmental Science Research Center, Faculty of Science Chiang Mai University Chiang Mai 50200 Thailand

^3^School of Allied Health Sciences and Research Institute for Health Sciences Walailak University Nakhon Si Thammarat 80160 Thailand

^4^Department of Chemistry, Faculty of Science Chiang Mai University Chiang Mai 50200 Thailand

^5^Department of Companion Animal and wildlife clinic, Faculty of Veterinary Medicine Chiang Mai University, Chiang Mai 50100 Thailand

^6^Department of Physics and Materials Science, Faculty of Science Chiang Mai University, Chiang Mai 50200 Thailand

^7^School of Science Walailak University, Nakhon Si Thammarat 80160 Thailand

^8^Environmental Science Research Center (ESRC), Faculty of Science Chiang Mai University, Chiang Mai 50200 Thailand

Correspondence and requests for materials should be addressed to C.R. (email: crandorn@gmail.com)


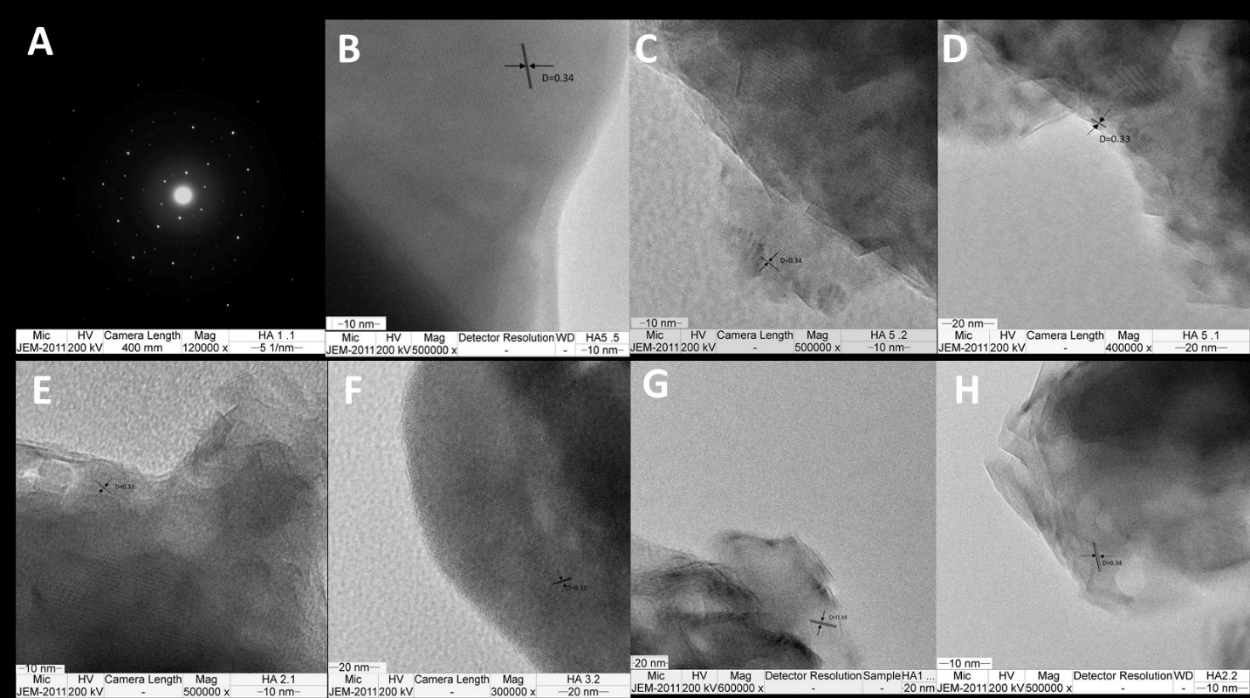


S1: images of (A) SAED of Microwave-HA, TEM images of (B) Commercial-HA and the synthesized HA after calcination at 600C : (C) Filtering-HA using H_2_O_2_, (D) Filtering-HA, (E) Microwave-HA using H_2_O_2_ , (F) Microwave-HA, (G) Hotplate-HA using H_2_O_2_ ,(H) Hotplate-HA


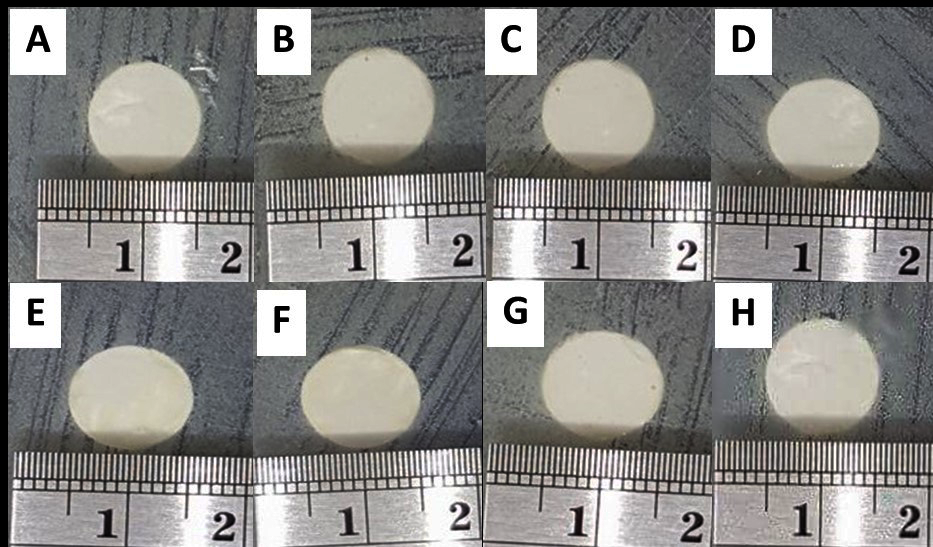


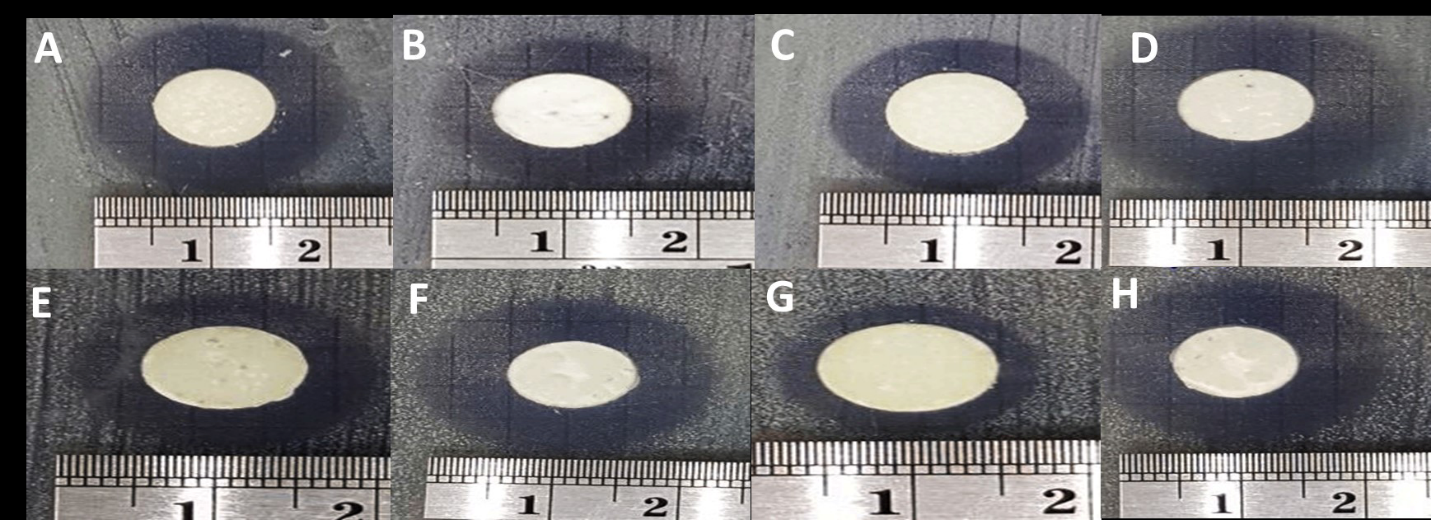
S2: images of antibacterial properties of HA after drying without combustion on E-coli : (A) Microwave-HA using H_2_O_2_, (B) Microwave-HA, (C) Hotplate-HA using H_2_O_2_, (D)Hotplate-HA and S-Aureus : (E) Microwave-HA using H_2_O_2_, (F) Microwave-HA, (G) Hotplate-HA using H_2_O_2_, (H) Hotplate-HA

S3: images of antibacterial properties of HA after drying with combustion on E-coli : (A) Microwave-HA using H_2_O_2_, (B) Microwave-HA, (C) Hotplate-HA using H_2_O_2_, (D)Hotplate-HA and S-Aureus : (E) Microwave-HA using H_2_O_2_, (F) Microwave-HA, (G) Hotplate-HA using H_2_O_2_, (H) Hotplate-HA
